# 2-[(3,4-Di­chloro­benzyl­idene)amino]-4-methyl­phenol

**DOI:** 10.1107/S1600536813013585

**Published:** 2013-05-25

**Authors:** Muhammet Kose, Ilyas Gonul, Vickie McKee

**Affiliations:** a Chemistry Department, K. Maras Sutcuimam University, 46100 K. Maras, Turkey; b Chemistry Department, Cukurova University, 01330, Turkey; c Chemistry Department, Loughborough University, Leicestershire LE11 3TU, England

## Abstract

In the title compound, C_14_H_11_Cl_2_NO, the dihedral angle between the benzene rings is 15.36 (8)°. A phenol–imine-type intra­molecular O—H⋯N hydrogen bond generates an *S*(5) ring motif. In the crystal, a pair of weak C—H⋯O hydrogen bonds form an *R*_2_^1^(7) ring motif involving glide-plane-related mol­ecules. The mol­ecules linked *via* these inter­actions form chains along [101].

## Related literature

For Schiff bases, see: Akine & Nabeshima (2009 ▶); Vigato & Tamburini (2004 ▶). For related structures, see: Efil *et al.* (2012 ▶); Fridman & Kaftory (2007 ▶); Jiao *et al.* (2006 ▶); Wang & Wang (2007 ▶). For hydrogen-bond motifs, see: Etter (1990 ▶); Bernstein *et al.* (1995 ▶).
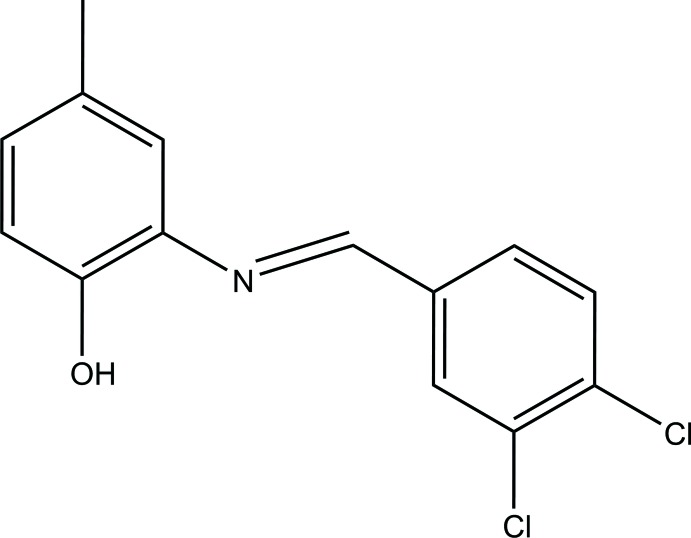


## Experimental

### 

#### Crystal data

C_14_H_11_Cl_2_NO
*M* *_r_* = 280.14
Monoclinic, 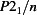

*a* = 4.6074 (7) Å
*b* = 21.680 (3) Å
*c* = 12.7907 (18) Å
β = 93.342 (2)°
*V* = 1275.5 (3) Å^3^
*Z* = 4
Mo *K*α radiation
μ = 0.49 mm^−1^
*T* = 150 K
0.64 × 0.14 × 0.07 mm

#### Data collection

Bruker APEXII CCD diffractometer
Absorption correction: multi-scan (*SADABS*; Sheldrick, 2009 ▶) *T*_min_ = 0.743, *T*_max_ = 0.966
12828 measured reflections
3159 independent reflections
2556 reflections with *I*2σ(*I*)
*R*_int_ = 0.032

#### Refinement

*R*[*F* ^2^ > 2σ(*F* ^2^)] = 0.035
*wR*(*F* ^2^) = 0.094
*S* = 1.02
3159 reflections
164 parameters
H-atom parameters constrained
Δρ_max_ = 0.35 e Å^−3^
Δρ_min_ = −0.23 e Å^−3^

### 

Data collection: *APEX2* (Bruker, 1998 ▶); cell refinement: *SAINT* (Bruker, 1998 ▶); data reduction: *SAINT*; program(s) used to solve structure: *SHELXS97* (Sheldrick, 2008 ▶); program(s) used to refine structure: *SHELXL97* (Sheldrick, 2008 ▶); molecular graphics: *ORTEP-3 for Windows* (Farrugia, 2012 ▶) and *Mercury* (Macrae *et al.*, 2008 ▶); software used to prepare material for publication: *SHELXTL* (Sheldrick, 2008 ▶).

## Supplementary Material

Crystal structure: contains datablock(s) I, global. DOI: 10.1107/S1600536813013585/fy2096sup1.cif

Structure factors: contains datablock(s) I. DOI: 10.1107/S1600536813013585/fy2096Isup2.hkl

Supplementary material file. DOI: 10.1107/S1600536813013585/fy2096Isup3.cml

Additional supplementary materials:  crystallographic information; 3D view; checkCIF report

## Figures and Tables

**Table 1:** Hydrogen-bond geometry (Å, °)

*D*—H⋯*A*	*D*—H	H⋯*A*	*D*⋯*A*	*D*—H⋯*A*
C5—H5⋯O1^i^	0.95	2.67	3.618 (2)	174
C8—H8⋯O1^i^	0.95	2.65	3.562 (2)	162
O1—H1⋯N1	0.81	2.16	2.6612 (17)	121

Symmetry code: (i) 
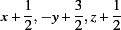
.

**Fig. 1.**
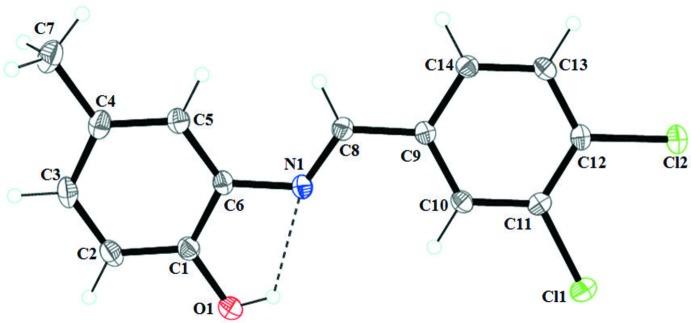
Structure of the title compound. Thermal ellipsoids are drawn at 30% probability and the intramolecular hydrogen bond is shown as a dashed line.

**Fig. 2.**
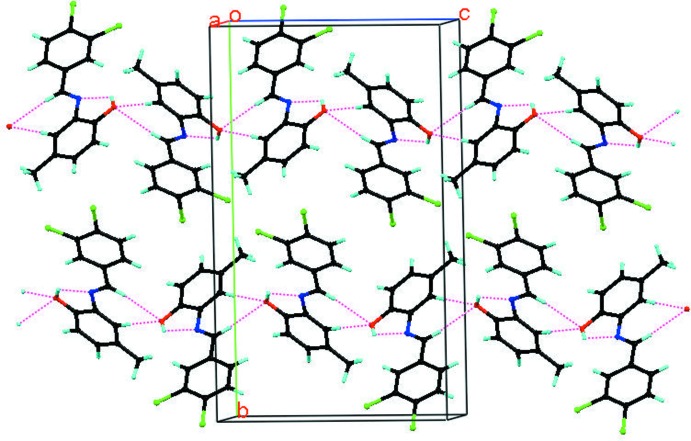
Intra- and intermolecular hydrogen bonding in the structure

**Fig. 3.**
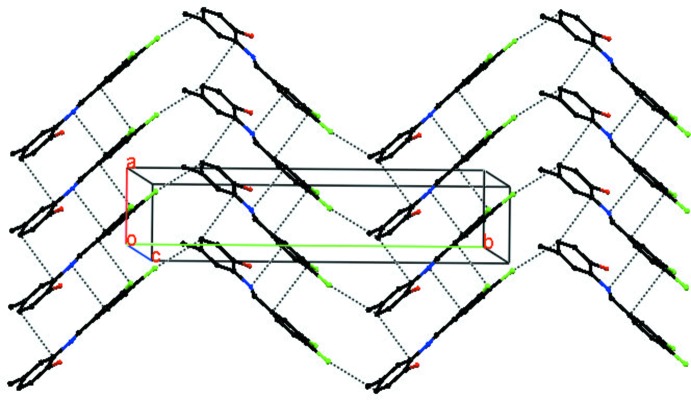
π-π and Cl-π interactions in the crystal of the title compound

## supplementary crystallographic information

### Comment

Schiff base condensations yield compounds with wide uses as ligands (Akine *et al.*, 2009; Vigato *et al.*, 2004). The title compound was prepared as a part of an investigation of the coordination and biological properties of Schiff base ligands.

The title compound adopts *E* configuration with respect to the imine C=N double bond with a C9—C8—N1—C6 torsion angle = -179.98 (13)°. The azomethine (C8═N1) bond distance is 1.2765 (19) Å and within the normal C═N values. The dihedral angle between the two benzene rings is 15.36 (8)°. There is a phenol-imine type intramolecular hydrogen bond (O1H···N1) in the structure forming a S(5) hydrogen bonding motif. There are two intermolecular weak hydrogen bond type (C5H···O1 and C8H···O1) interactions, resulting in a R^1^_2_(7) hydrogen-bonding motif. Molecules are linked *via* weak hydrogen bonding form hydrogen-bond chains along the *ac* diagonal (Fig. 2, Table 1).

There is evidence of π-π stacking in the structure. The C3—C9 section of the molecule is stacked with the C6*—C12* section of an adjacent molecule (* = *x* + 1, *y*, *z*). N1 and C10* are seperated by a distance of 3.302 (2) Å. There is also π-Cl interaction in the structure: Cl2 is stacked with C3** of an adjacent molecule (** = 1/2 - *x*, 1/2 + *y*, 3/2 - *z*) with a distance of 3.407 (2) Å (Fig. 3).

### Experimental

A solution of 3,4-dichlorobenzaldehyde (0.0.525 g, 3 mmol) in methanol (25 ml) was added to a methanolic solution (20 ml) of 2-amino-4-methylphenol (0.740 g, 6 mmol). The mixture was stirred for two hours at room temperature and left for air evaporation. After two days, yellow crystals suitable for X-ray diffraction study were collected by filtration.

### Refinement

H atoms bonded to C were inserted at calculated positions with C—H distances of 0.95 and 0.99 Å for non-saturated and saturated C atoms, respectively, They were refined using a riding model with *U*_iso_(H) = 1.2 U_eq_(C). The H-atom bonded to O1 was taken directly from the difference Fourier map and was refined with a riding model using temperature factors *U*_iso_(H) = 1.5 U_eq_(O).

### Crystal data

**Table:**

C_14_H_11_Cl_2_NO	*F*(000) = 576
*M**_r_* = 280.14	*D*_x_ = 1.459 Mg m^−^^3^
Monoclinic, *P*2_1_/*n*	Mo *K*α radiation, λ = 0.71073 Å
Hall symbol: -P 2yn	Cell parameters from 3681 reflections
*a* = 4.6074 (7) Å	θ = 2.5–28.0°
*b* = 21.680 (3) Å	µ = 0.49 mm^−^^1^
*c* = 12.7907 (18) Å	*T* = 150 K
β = 93.342 (2)°	Block, yellow
*V* = 1275.5 (3) Å^3^	0.64 × 0.14 × 0.07 mm
*Z* = 4	

### Data collection

**Table:**

Bruker APEXII CCD diffractometer	3159 independent reflections
Radiation source: fine-focus sealed tube	2556 reflections with *I*2σ(*I*)
Graphite monochromator	*R*_int_ = 0.032
ω rotation with narrow frames scans	θ_max_ = 28.3°, θ_min_ = 1.9°
Absorption correction: multi-scan (*SADABS*; Sheldrick, 2009)	*h* = −6→6
*T*_min_ = 0.743, *T*_max_ = 0.966	*k* = −28→28
12828 measured reflections	*l* = −17→16

### Refinement

**Table:**

Refinement on *F*^2^	Primary atom site location: structure-invariant direct methods
Least-squares matrix: full	Secondary atom site location: difference Fourier map
*R*[*F*^2^ > 2σ(*F*^2^)] = 0.035	Hydrogen site location: inferred from neighbouring sites
*wR*(*F*^2^) = 0.094	H-atom parameters constrained
*S* = 1.02	*w* = 1/[σ^2^(*F*_o_^2^) + (0.0503*P*)^2^ + 0.3141*P*] where *P* = (*F*_o_^2^ + 2*F*_c_^2^)/3
3159 reflections	(Δ/σ)_max_ = 0.001
164 parameters	Δρ_max_ = 0.35 e Å^−^^3^
0 restraints	Δρ_min_ = −0.23 e Å^−^^3^

### Special details

**Table:**

Geometry. All e.s.d.'s (except the e.s.d. in the dihedral angle between two l.s. planes) are estimated using the full covariance matrix. The cell e.s.d.'s are taken into account individually in the estimation of e.s.d.'s in distances, angles and torsion angles; correlations between e.s.d.'s in cell parameters are only used when they are defined by crystal symmetry. An approximate (isotropic) treatment of cell e.s.d.'s is used for estimating e.s.d.'s involving l.s. planes.
Refinement. Refinement of *F*^2^ against ALL reflections. The weighted *R*-factor *wR* and goodness of fit *S* are based on *F*^2^, conventional *R*-factors *R* are based on *F*, with *F* set to zero for negative *F*^2^. The threshold expression of *F*^2^ > σ(*F*^2^) is used only for calculating *R*-factors(gt) *etc*. and is not relevant to the choice of reflections for refinement. *R*-factors based on *F*^2^ are statistically about twice as large as those based on *F*, and *R*- factors based on ALL data will be even larger.

### Fractional atomic coordinates and isotropic or equivalent isotropic displacement parameters (Å^2^)

**Table:**

	*x*	*y*	*z*	*U*_iso_*/*U*_eq_	
Cl1	0.62127 (9)	0.968261 (19)	0.61947 (3)	0.03521 (12)	
N1	−0.1214 (3)	0.79478 (6)	0.73732 (10)	0.0234 (3)	
O1	−0.4296 (3)	0.77697 (6)	0.55736 (9)	0.0381 (3)	
H1	−0.2991	0.7997	0.5779	0.057*	
C1	−0.4801 (3)	0.73837 (7)	0.63921 (12)	0.0264 (3)	
Cl2	0.86902 (8)	1.020614 (17)	0.83841 (3)	0.03076 (12)	
C2	−0.6873 (4)	0.69200 (8)	0.62554 (13)	0.0321 (4)	
H2	−0.7926	0.6872	0.5600	0.039*	
C3	−0.7391 (3)	0.65297 (7)	0.70787 (14)	0.0322 (4)	
H3	−0.8816	0.6215	0.6982	0.039*	
C4	−0.5859 (3)	0.65887 (7)	0.80533 (13)	0.0297 (3)	
C5	−0.3799 (3)	0.70580 (7)	0.81783 (12)	0.0261 (3)	
H5	−0.2752	0.7107	0.8835	0.031*	
C6	−0.3245 (3)	0.74577 (7)	0.73566 (11)	0.0228 (3)	
C7	−0.6423 (4)	0.61546 (8)	0.89403 (15)	0.0406 (4)	
H7A	−0.5103	0.6252	0.9546	0.061*	
H7B	−0.6092	0.5729	0.8718	0.061*	
H7C	−0.8439	0.6200	0.9133	0.061*	
C8	0.0181 (3)	0.81112 (7)	0.82195 (12)	0.0235 (3)	
H8	−0.0153	0.7899	0.8851	0.028*	
C9	0.2291 (3)	0.86195 (7)	0.82415 (11)	0.0216 (3)	
C10	0.3150 (3)	0.88903 (7)	0.73156 (12)	0.0233 (3)	
H10	0.2364	0.8745	0.6658	0.028*	
C11	0.5146 (3)	0.93697 (7)	0.73568 (11)	0.0235 (3)	
C12	0.6295 (3)	0.95870 (7)	0.83241 (12)	0.0233 (3)	
C13	0.5479 (3)	0.93173 (7)	0.92445 (12)	0.0245 (3)	
H13	0.6282	0.9461	0.9901	0.029*	
C14	0.3485 (3)	0.88372 (7)	0.92030 (12)	0.0239 (3)	
H14	0.2924	0.8654	0.9835	0.029*	

### Atomic displacement parameters (Å^2^)

**Table:**

	*U*^11^	*U*^22^	*U*^33^	*U*^12^	*U*^13^	*U*^23^
Cl1	0.0427 (3)	0.0358 (2)	0.0276 (2)	−0.00705 (17)	0.00626 (17)	0.00436 (15)
N1	0.0204 (6)	0.0227 (6)	0.0268 (6)	−0.0010 (5)	−0.0001 (5)	−0.0005 (5)
O1	0.0430 (7)	0.0457 (7)	0.0249 (6)	−0.0154 (6)	−0.0044 (5)	0.0008 (5)
C1	0.0253 (8)	0.0272 (8)	0.0270 (8)	−0.0017 (6)	0.0029 (6)	−0.0035 (6)
Cl2	0.0272 (2)	0.0259 (2)	0.0390 (2)	−0.00604 (14)	0.00070 (15)	−0.00215 (15)
C2	0.0267 (8)	0.0361 (9)	0.0332 (9)	−0.0049 (7)	−0.0002 (6)	−0.0109 (7)
C3	0.0243 (8)	0.0262 (8)	0.0466 (10)	−0.0060 (6)	0.0076 (7)	−0.0110 (7)
C4	0.0276 (8)	0.0223 (7)	0.0403 (9)	−0.0003 (6)	0.0102 (7)	−0.0023 (6)
C5	0.0247 (7)	0.0237 (7)	0.0299 (8)	0.0007 (6)	0.0016 (6)	−0.0017 (6)
C6	0.0188 (7)	0.0219 (7)	0.0279 (8)	−0.0002 (5)	0.0026 (6)	−0.0040 (6)
C7	0.0455 (11)	0.0269 (9)	0.0508 (11)	−0.0042 (7)	0.0135 (9)	0.0033 (8)
C8	0.0206 (7)	0.0239 (7)	0.0258 (7)	0.0006 (5)	0.0007 (5)	0.0012 (6)
C9	0.0179 (7)	0.0214 (7)	0.0252 (7)	0.0024 (5)	−0.0003 (5)	−0.0003 (5)
C10	0.0219 (7)	0.0239 (7)	0.0237 (7)	0.0016 (5)	−0.0018 (5)	−0.0020 (6)
C11	0.0234 (7)	0.0228 (7)	0.0246 (7)	0.0037 (6)	0.0037 (6)	0.0027 (6)
C12	0.0187 (7)	0.0199 (7)	0.0311 (8)	0.0011 (5)	0.0003 (6)	−0.0012 (6)
C13	0.0228 (7)	0.0260 (7)	0.0241 (7)	0.0015 (6)	−0.0031 (6)	−0.0027 (6)
C14	0.0219 (7)	0.0260 (7)	0.0234 (7)	0.0017 (6)	−0.0014 (5)	0.0020 (6)

### Geometric parameters (Å, º)

**Table:**

Cl1—C11	1.7310 (15)	C5—H5	0.9500
N1—C8	1.2766 (19)	C7—H7A	0.9800
N1—C6	1.4153 (19)	C7—H7B	0.9800
O1—C1	1.3708 (19)	C7—H7C	0.9800
O1—H1	0.8089	C8—C9	1.469 (2)
C1—C2	1.390 (2)	C8—H8	0.9500
C1—C6	1.399 (2)	C9—C10	1.399 (2)
Cl2—C12	1.7366 (15)	C9—C14	1.400 (2)
C2—C3	1.382 (2)	C10—C11	1.387 (2)
C2—H2	0.9500	C10—H10	0.9500
C3—C4	1.402 (2)	C11—C12	1.399 (2)
C3—H3	0.9500	C12—C13	1.386 (2)
C4—C5	1.394 (2)	C13—C14	1.387 (2)
C4—C7	1.508 (2)	C13—H13	0.9500
C5—C6	1.397 (2)	C14—H14	0.9500
			
C8—N1—C6	121.40 (13)	H7A—C7—H7C	109.5
C1—O1—H1	106.3	H7B—C7—H7C	109.5
O1—C1—C2	119.44 (14)	N1—C8—C9	121.64 (14)
O1—C1—C6	120.14 (13)	N1—C8—H8	119.2
C2—C1—C6	120.42 (15)	C9—C8—H8	119.2
C3—C2—C1	119.65 (15)	C10—C9—C14	119.09 (13)
C3—C2—H2	120.2	C10—C9—C8	121.20 (13)
C1—C2—H2	120.2	C14—C9—C8	119.71 (13)
C2—C3—C4	121.39 (15)	C11—C10—C9	120.12 (13)
C2—C3—H3	119.3	C11—C10—H10	119.9
C4—C3—H3	119.3	C9—C10—H10	119.9
C5—C4—C3	118.21 (15)	N1^i^—C10—H10	94.2
C5—C4—C7	121.01 (16)	C10—C11—C12	120.13 (14)
C3—C4—C7	120.79 (15)	C10—C11—Cl1	118.82 (11)
C4—C5—C6	121.28 (15)	C12—C11—Cl1	121.04 (12)
C4—C5—H5	119.4	C13—C12—C11	120.12 (14)
C6—C5—H5	119.4	C13—C12—Cl2	119.45 (11)
C5—C6—C1	119.04 (14)	C11—C12—Cl2	120.41 (12)
C5—C6—N1	127.15 (13)	C12—C13—C14	119.73 (14)
C1—C6—N1	113.81 (13)	C12—C13—H13	120.1
C4—C7—H7A	109.5	C14—C13—H13	120.1
C4—C7—H7B	109.5	C13—C14—C9	120.80 (14)
H7A—C7—H7B	109.5	C13—C14—H14	119.6
C4—C7—H7C	109.5	C9—C14—H14	119.6
			
O1—C1—C2—C3	−179.97 (15)	N1—C8—C9—C10	−8.8 (2)
C6—C1—C2—C3	−0.1 (2)	N1—C8—C9—C14	171.57 (14)
C1—C2—C3—C4	−0.3 (2)	C14—C9—C10—C11	−0.3 (2)
C2—C3—C4—C5	0.6 (2)	C8—C9—C10—C11	180.00 (13)
C2—C3—C4—C7	−179.26 (15)	C9—C10—C11—C12	−0.4 (2)
C3—C4—C5—C6	−0.5 (2)	C9—C10—C11—Cl1	178.69 (11)
C7—C4—C5—C6	179.34 (15)	C10—C11—C12—C13	1.1 (2)
C4—C5—C6—C1	0.1 (2)	Cl1—C11—C12—C13	−178.00 (11)
C4—C5—C6—N1	−179.53 (14)	C10—C11—C12—Cl2	−177.57 (11)
O1—C1—C6—C5	−179.95 (14)	Cl1—C11—C12—Cl2	3.38 (18)
C2—C1—C6—C5	0.2 (2)	C11—C12—C13—C14	−1.0 (2)
O1—C1—C6—N1	−0.2 (2)	Cl2—C12—C13—C14	177.67 (11)
C2—C1—C6—N1	179.88 (14)	C12—C13—C14—C9	0.2 (2)
C8—N1—C6—C5	−6.9 (2)	C10—C9—C14—C13	0.4 (2)
C8—N1—C6—C1	173.46 (14)	C8—C9—C14—C13	−179.91 (13)
C6—N1—C8—C9	−179.98 (13)		

Symmetry code: (i) *x*+1, *y*, *z*.

### Hydrogen-bond geometry (Å, º)

**Table:**

*D*—H···*A*	*D*—H	H···*A*	*D*···*A*	*D*—H···*A*
C5—H5···O1^ii^	0.95	2.67	3.618 (2)	174
C8—H8···O1^ii^	0.95	2.65	3.562 (2)	162
O1—H1···N1	0.81	2.16	2.6612 (17)	121

Symmetry code: (ii) *x*+1/2, −*y*+3/2, *z*+1/2.

## References

[bb1] Akine, S. & Nabeshima, T. (2009). *Dalton Trans.* pp. 10395–10408. 10.1039/b910989g 20023858

[bb2] Bernstein, J., Davis, R. E., Shimoni, L. & Chang, N.-L. (1995). *Angew. Chem. Int. Ed. Engl.* **34**, 1555–1573.

[bb3] Bruker (1998). *APEX2* and *SAINT* Bruker AXS Inc., Madison, Wisconsin, USA.

[bb4] Efil, K., Şen, F., Bekdemir, Y. & Büyükgüngör, O. (2012). *Acta Cryst.* E**68**, o1696. 10.1107/S1600536812019770 PMC3379291 22719489

[bb5] Etter, M. C. (1990). *Acc. Chem. Res.* **23**, 120–126.

[bb6] Farrugia, L. J. (2012). *J. Appl. Cryst.* **45**, 849–854.

[bb7] Fridman, N. & Kaftory, M. (2007). *Pol. J. Chem.* **81**, 825–832.

[bb8] Jiao, Y.-H., Zhang, Q. & Ng, S. W. (2006). *Acta Cryst.* E**62**, o3614–o3615.

[bb9] Macrae, C. F., Bruno, I. J., Chisholm, J. A., Edgington, P. R., McCabe, P., Pidcock, E., Rodriguez-Monge, L., Taylor, R., van de Streek, J. & Wood, P. A. (2008). *J. Appl. Cryst.* **41**, 466–470.

[bb10] Sheldrick, G. M. (2008). *Acta Cryst.* A**64**, 112–122. 10.1107/S0108767307043930 18156677

[bb11] Sheldrick, G. M. (2009). *SADABS* University of Göttingen, Germany.

[bb12] Vigato, P. A. & Tamburini, S. (2004). *Coord. Chem. Rev.* **248**, 1717–2128.

[bb13] Wang, Q. & Wang, D.-Q. (2007). *Acta Cryst.* E**63**, o4838.

